# Hepatoid adenocarcinoma of the lung: clinicopathologic and molecular analysis of 17 cases

**DOI:** 10.1007/s00428-026-04558-3

**Published:** 2026-05-07

**Authors:** Behtash G. Nezami, Stanislav Fridland, Lucas Santana dos Santos, Lawrence Jennings, Madina Sukhanova, Xinyan Lu, Ankit Bharat, Samuel S. Kim, David Dittman, Vikas Mehta, Anjana Yeldandi, Borislav A. Alexiev

**Affiliations:** 1 Department of Pathology, Northwestern University Feinberg School of Medicine, Chicago, IL USA; 2 Department of Thoracic Surgery, Northwestern University Feinberg School of Medicine, Chicago, IL USA

**Keywords:** Hepatoid adenocarcinoma, Lung adenocarcinoma, Hepatoid differentiation, Immunohistochemistry, Molecular pathology

## Abstract

Hepatoid adenocarcinoma of the lung (HAL) is a rare subtype of non–small cell lung carcinoma characterized by morphologic and immunophenotypic overlap with hepatocellular carcinoma (HCC), creating diagnostic challenges for practicing pathologists. Because of its rarity, only limited information exists regarding its molecular profile and biologic behavior. We retrospectively analyzed 17 HAL cases diagnosed at Northwestern Memorial Hospital and describe their clinical presentation, morphologic features, immunohistochemical and molecular profiles, and clinical outcomes. Histologically, HAL is composed of polygonal to columnar epithelioid cells with abundant lightly eosinophilic, finely granular cytoplasm and predominantly solid and/or complex glandular growth patterns. Foci of mucinous differentiation may be present. Cytologic atypia, brisk mitotic activity, and tumor necrosis are common findings. Immunohistochemically, HAL is characterized by strong and diffuse HepPar1 expression, positivity for adenocarcinoma markers such as MOC31, and cytoplasmic immunoreactivity for TTF-1. Across the 17 cases, *STK11* alterations were most frequent (52.9%), followed by *TP53* (41.2%) and *KRAS* (35.3%). Concurrent *KRAS* + *STK11* alterations were identified in 23.5% of cases, compared with The Cancer Genome Atlas (TCGA) cohorts of other lung adenocarcinomas (11/566, 2.0%) and HCC (0/372, 0%). HAL demonstrates highly aggressive behavior, with metastatic presentation even in low-stage tumors (T1: 33.3%; T2: 71.4%), frequent hematogenous spread, and death of disease in 41.2% of patients at a median follow-up of 5 months. In summary, HAL is a distinct subtype with characteristic morphologic and immunohistochemical features. Given its aggressive clinical course, accurate diagnosis is essential for patient management.

**Supplementary Information:**

The online version contains supplementary material available at 10.1007/s00428-026-04558-3.

## Introduction

Hepatoid adenocarcinoma of the lung (HAL) is a rare subtype of non–small cell lung carcinoma (NSCLC) in which the principal diagnostic challenge stems from its morphologic and immunophenotypic overlap with hepatocellular carcinoma (HCC). Early descriptions focused on AFP-producing primary lung carcinomas in patients without underlying liver disease, and Ishikura et al. subsequently introduced *hepatoid adenocarcinoma* as a histologic category defined by hepatocellular-like morphology accompanied by hepatic marker expression [1–4]. In contemporary practice, the term is applied pragmatically to tumors that demonstrate convincing hepatoid morphology and hepatic marker expression after exclusion of metastases [5–10]. This distinction is particularly consequential in patients presenting with synchronous lung and liver lesions, where resolving the HAL–HCC differential requires integrated radiologic, morphologic, and immunophenotypic assessment.

Despite increasing recognition, several limitations across published cohorts continue to constrain biologic and clinical interpretation. First, HAL is widely regarded as aggressive, largely because many reported cases present with metastatic disease and have poor outcomes [11–13]. However, these impressions are derived from small series that are susceptible to referral patterns and stage-enrichment biases [14–16]. Second, HAL may exhibit histological features that are atypical for HCC and not well appreciated by practicing pathologists, reducing diagnostic accuracy in challenging cases. Third, molecular characterization remains limited. Earlier cohorts predated comprehensive next-generation sequencing (NGS), and more recent studies emphasize the absence of “targetable” alterations or report isolated gene frequencies [14, 15, 17, 18]. As a result, it remains unclear whether the poor outcomes reflect biased sampling of advanced-stage tumors or whether HAL is consistently enriched for high-risk lung adenocarcinoma (LUAD) genomic profiles.

To address these gaps, we analyzed 17 cases of HAL diagnosed in our pathology department and investigated the issue using three complementary approaches.

First, we examine the HAL–HCC differential through the lens of routine diagnostic workflows, integrating the morphologic features and immunophenotypic panels used in actual cases with literature-based marker recommendations [5]. Second, we move beyond stage-at-presentation as a surrogate for aggressiveness by quantifying metastatic involvement conditional on primary tumor burden. Third, we define a LUAD-relevant genomic architecture—capturing both mutational landscape and co-alteration patterns—to contextualize HAL’s clinical behavior within established lung adenocarcinoma biology [14, 15].

## Methods

### Ethics, study design, and case identification

This retrospective single-institution study of hepatoid adenocarcinoma of the lung (HAL), including external consultation material, was approved by the Institutional Review Board of Northwestern University (IRB #STU00210244) with waiver of consent. Cases diagnosed between 2023 and 2025 were identified through institutional pathology archives and molecular pathology records. Because molecular testing is routine for lung adenocarcinomas at our institution, this approach is unlikely to introduce meaningful enrichment bias.

### Case definition, pathologic review, and histopathologic assessment

Cases were retrieved from institutional archives and centrally reviewed by three pathologists (BA, AY, VM). HAL was defined by pulmonary primary context, convincing hepatoid morphology on H&E (solid or trabecular growth pattern with cells exhibiting abundant granular and/or clear/vacuolated cytoplasm), and immunophenotypic support for hepatoid differentiation (HepPar1 expression), with exclusion of concurrent HCC or evidence of metastasis from an active non-pulmonary primary at diagnosis.

### Histology

Histologic patterns were recorded using standard lung adenocarcinoma terminology. Additional features, including complex glands, necrosis, and squamous differentiation, were documented when present. In resection specimens, mixed histology was recorded when multiple patterns were identified; in biopsy-only cases, pattern assessment was interpreted in the context of sampling limitations. Representative sections were fixed in 10% buffered formalin, embedded in paraffin, and stained with hematoxylin and eosin on 4-µm sections.

### Immunohistochemistry

A full description of our immunohistochemistry methods can be found in the Supplemental Material.

### Staging and metastatic-at-diagnosis endpoint

For the institutional HAL cohort, clinical and pathologic TNM stage was assigned per AJCC/UICC 8th edition using contemporaneous imaging and pathology reports (biopsy-only cases: cTNM; resections: pTNM when applicable). For stage-conditional analyses, best-available T category was used, and M status was recorded at presentation. For M1 cases, metastatic organ-site burden was summarized as the number of distinct distant organ-site categories at diagnosis (bone, adrenal, liver, brain, kidney, lung); lymph node involvement was recorded separately.

### Comparator cohorts

A full description of the Surveillance, Epidemiology, and End Results (SEER) and The Cancer Genome Atlas (TCGA) LUAD cohorts can be found in the Supplemental Material.

### Molecular profiling and genomic annotation

Cases underwent targeted sequencing with the PGDx™ elio™ tissue complete assay performed per manufacturer and institutional clinical workflows. One case was tested using an alternative clinically validated targeted assay (Oncomine Precision Assay). A detailed description of the molecular methods is provided in the Supplemental Material.

### Comparative oncoplot construction and co-alteration definitions

For cross-cohort comparison, a HAL-derived gene list was defined as genes mutated in ≥ 2 HAL cases and displayed in an oncoplot. TCGA LUAD and TCGA HCC mutation data were retrieved via cBioPortal. TCGA variants were filtered to OncoKB “oncogenic” or “likely oncogenic” calls. Alterations were grouped into broad mutation classes (missense, truncating, splice, inframe).

### Statistical analysis

A full description of our statistical methods can be found in the Supplemental Material.

## Results

### Cohort and clinicopathologic features

Seventeen patients met study inclusion criteria (Table 1; Tables S1–S2), comprising 7 resections and 10 biopsy-only cases. The median age at diagnosis was 69 years (range, 50.8–87.3), 7 patients were male (41.2%), and 77% had a smoking history (median 41 pack-years). The median primary tumor size was 4.5 cm (range, 1.2–7.8 cm). Clinical stage at presentation was I in 3/17 (17.6%), III in 5/17 (29.4%), and IV in 9/17 (52.9%).

**Table 1:** Clinicopathologic characteristics, metastatic patterns, and clinical outcomes in a 17-case cohort of hepatoid adenocarcinoma of the lung

Characteristics	Range
Median age, years (range)	69 (50.8–87.3)
Male, n (%)	7 (41.2%)
History of smoking, n (%)	13 (77%)
Median pack-years (range)	41 (8-100)
Primary site^b c^	
Right upper lobe, n (%)	5 (29%)
Right middle lobe, n (%)	4 (24%)
Right lower lobe, n (%)	3 (17%)
Left upper lobe, n (%)	4 (24%)
Left lower lobe, n (%)	1 (6%)
Median primary tumor size, cm (range)	4.5 (1.2–7.8)
Clinical Stage^a b^, n (%)	
I	3 (18%)
II	0 (0%)
III	5 (29%)
IV	9 (53%)
Pathologic Stage^a b^, n (%)	
T1, T2, T3, T4	3 (17.6), 7 (41.2), 2 (11.8), 5 (29.4)
N0, N1, N2, N3, Nx	4 (23.5), 4 (23.5), 7 (41.2), 1 (5.9), 1 (5.9)
M0, M1, Mx	1 (5.9), 9 (52.9), 7 (41.2)
Median metastatic organ sites at diagnosis^d^ (range)	3 (2–5)
Bone involvement, n (%)	7 (41.2%)
Adrenal involvement, n (%)	4 (23.5%)
Liver involvement, n (%)	2 (11.8%)
Brain involvement, n (%)	1 (5.9%)
Vital status at last follow-up^b^, n (%)	
NED	4 (23.5%)
AWD	3 (17.6%)
DOD	10 (58.8%)
Median follow-up, months (range)	5 (0.5–103)
Median OS, months	
Overall	6
Stage IV only	3

a Stage is reported as pathologic stage for resection specimens and clinical stage for biopsy-only specimens, according to the AJCC/UICC 8th edition staging system

b Percentages are calculated using the total cohort size (*n* = 17) unless otherwise specified

c Some tumors involved multiple lobes, primary site refers to the lobe with the largest mass

d Metastatic organ-site data are reported for stage IV/M1 cases only (*n* = 9), and percentages for individual organ sites use these 9 cases as the denominator. Organ-site categories are not mutually exclusive because some patients had metastases involving multiple anatomic sites at diagnosis

Abbreviations: *HAL* hepatoid adenocarcinoma of the lung, *NED* no evidence of disease, *AWD* alive with disease, *DOD* died of disease, *OS* overall survival, *AJCC* American Joint Committee on Cancer, *UICC* Union for International Cancer Control,* M1* distant metastasis present at diagnosis

Grossly, resected tumors were bulky, tan-yellow, and poorly circumscribed. Figure S1 illustrates a representative example in which the cut surface shows a variegated, soft, friable mass with interspersed hemorrhage and necrosis, extending to the pleural surface without a discrete capsule and contrasting with the relatively unremarkable background lung parenchyma.

**Fig. 1:**
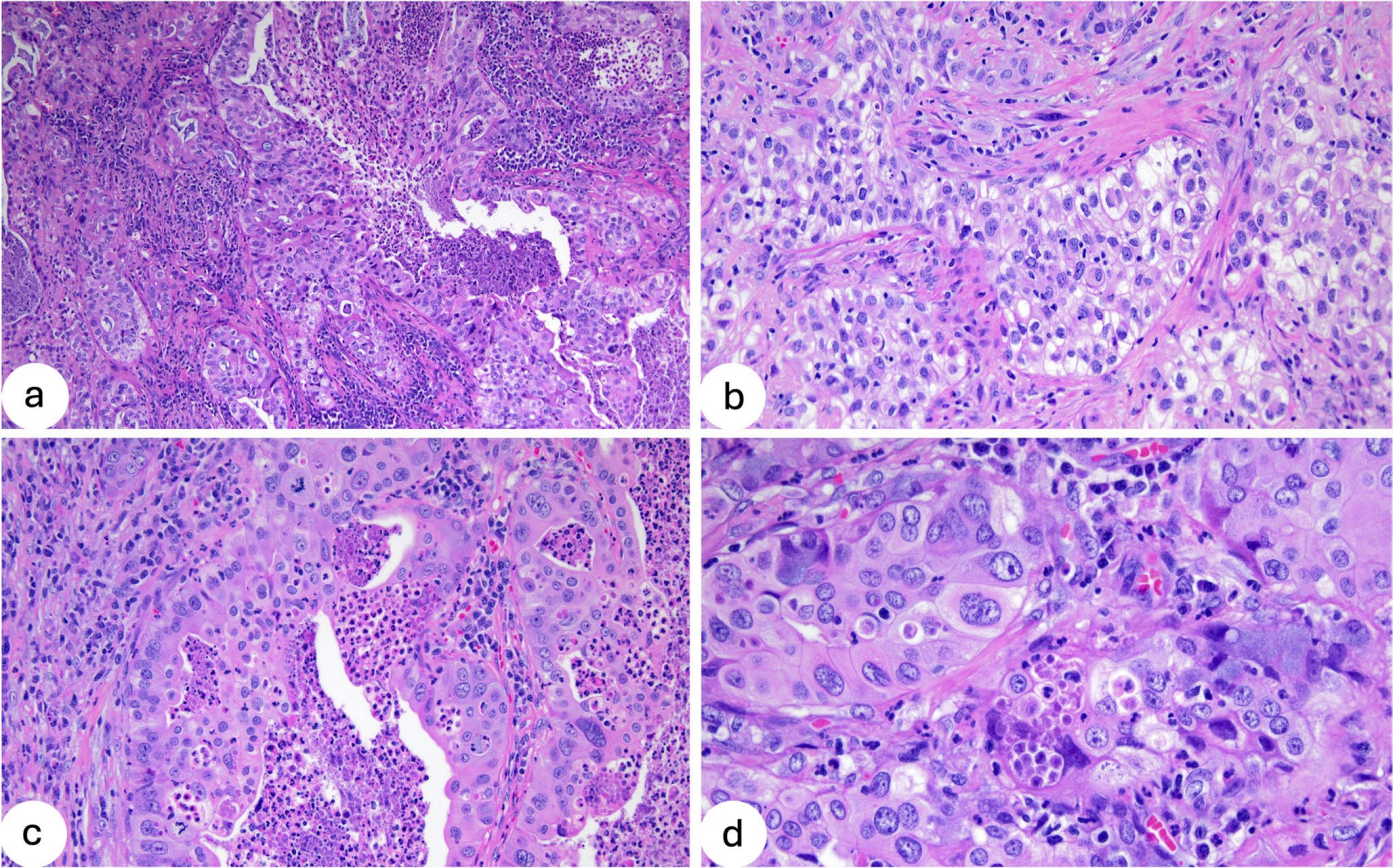
Histologic features of hepatoid adenocarcinoma of the lung (**A**) Light microscopic examination demonstrates a complex glandular pattern (Hematoxylin-eosin, 100x). (**B**) Light microscopic examination demonstrates a solid growth pattern (Hematoxylin-eosin, 200x). (**C**) Light microscopic examination demonstrates mitotic activity and tumor necrosis (Hematoxylin-eosin, 200x). (**D**) Light microscopic examination demonstrates tumor cells with abundant eosinophilic cytoplasm and nuclei displaying vesicular chromatin. Scattered cells contain intracytoplasmic mucin. (Hematoxylin-eosin, 200x)

On hematoxylin and eosin sections (Fig. 1A–D), the tumors were composed of polygonal to columnar cells with distinct borders, abundant lightly eosinophilic finely granular cytoplasm, and round to ovoid nuclei with vesicular chromatin and prominent nucleoli. Across the cohort, tumors demonstrated predominantly a solid growth pattern (12/17, 70.6%). Complex glandular architecture was present in 7/17 tumors (41.2%). Acinar differentiation was common (9/17, 52.9%), whereas papillary (4/17, 23.5%), lepidic (4/17, 23.5%), micropapillary (3/17, 17.6%), and mucinous (4/17, 23.5%) components were less frequent. Mitoses were readily identified, tumor necrosis occurred in 7/17 (41.2%), and squamous differentiation was rare (1/17, 5.9%).

**Fig. 2:**
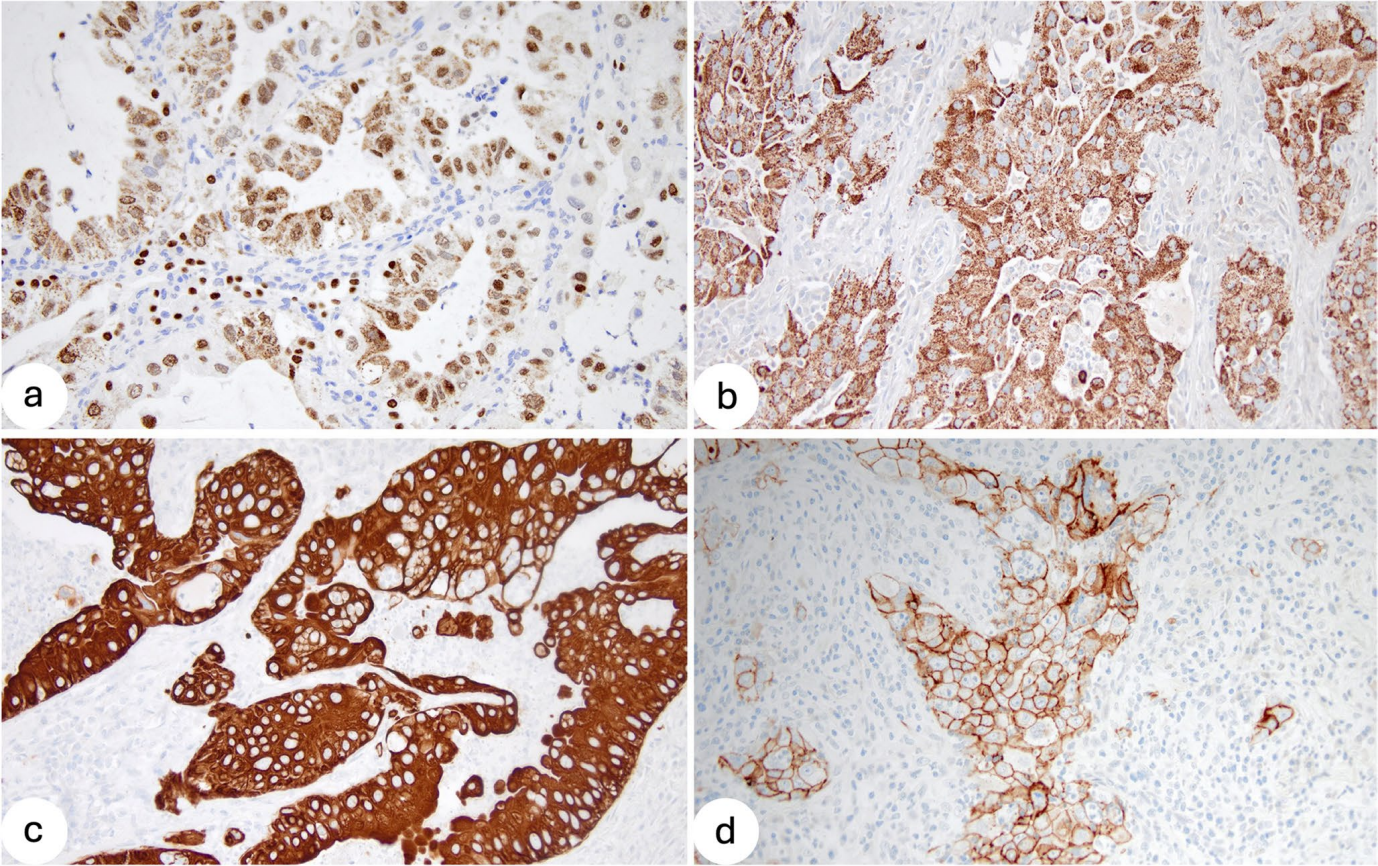
Immunohistochemical features of hepatoid adenocarcinoma of the lung (**A**) Predominantly cytoplasmic positivity for TTF-1 in tumor cells (TTF-1, 200x). (**B**) HepPar1 positivity in tumor cells (HepPar1, 200x). (**C**) CK7 positivity in tumor cells (CK7, 200x). (**D**) MOC31 positivity in tumor cells (MOC31, 200x)

Immunohistochemical findings are summarized in Fig. 2A–D and Table S3. HepPar1 was positive in all cases (17/17, 100%). TTF-1 staining was present in 16/17 cases (94.1%; one weak), with characteristic cytoplasmic immunoreactivity in tumor cells. Cytokeratin expression was detected in all tested tumors (CK7 positive in 12 cases and CK AE1/AE3 positive in 2 additional, non-overlapping cases); 3 cases lacked cytokeratin testing. MOC31 was uniformly positive (16/16, 100%). Napsin A was positive in 3/11 cases (27.3%). p40 was negative in 7/8 cases (87.5%), with focal or subset positivity in one case.

PD-L1 tumor proportion score (TPS) was < 1% in 7/17 (41.2%), 1–49% in 8/17 (47.1%), and ≥ 50% in 2/17 (11.8%).

### Genomic architecture and cross-cohort oncoplot comparison

Across the 17 HAL cases, *STK11* alterations were most frequent (9/17, 52.9%; predominantly truncating or splice-site variants), followed by *TP53* (7/17, 41.2%) and *KRAS* (6/17, 35.3%). *KRAS* mutations were enriched for canonical hotspot substitutions (G12C 3/6, G12D 2/6, G12V 1/6). Additional recurrent alterations included *KEAP1* (3/17, 17.6%), *SMARCA4* (3/17, 17.6%), *CDKN2A* (2/17, 11.8%), and *LRP1B* (2/17, 11.8%). A case-level summary of all molecular alterations is provided in Table S4.

**Fig. 3:**
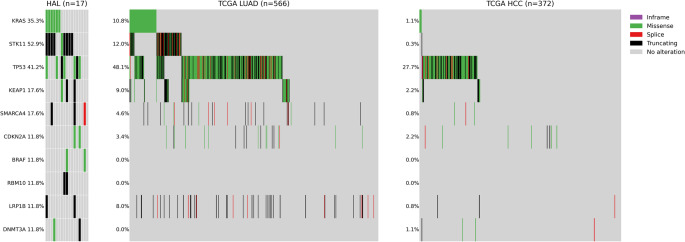
Cross-cohort oncoplot comparison of recurrent genomic alterations in hepatoid adenocarcinoma of the lung (HAL) and reference TCGA cohorts. Oncoplots display somatic alterations across HAL (*n* = 17), TCGA lung adenocarcinoma (LUAD; *n* = 566), and TCGA hepatocellular carcinoma (HCC; *n* = 372). Genes shown were restricted to those altered in ≥ 2 HAL cases (left axis). Percentages present at the left axis of each plot represent the prevalence of mutations within the respective gene within each cohort. For TCGA cohorts, displayed variants were filtered to OncoKB “oncogenic” or “likely oncogenic” calls. Color indicates alteration class (inframe, missense, splice, truncating), with gray denoting no “oncogenic” or “likely oncogenic” alteration. Percentages at left of each cohort indicate the proportion of cases altered for each gene within that cohort

Within HAL, *STK11* was the dominant recurrently altered gene (9/17, 52.9%), exceeding TCGA LUAD (12.0%) and TCGA HCC (0.3%). *KRAS* alterations were enriched in HAL (6/17, 35.3%) relative to TCGA LUAD (10.8%) and TCGA HCC (1.1%). *TP53* alterations occurred in 7/17 HAL (41.2%), higher than TCGA HCC (27.7%) but lower than TCGA LUAD (48.1%). Concurrent *KRAS*+*STK11* alterations were present in 4/17 HAL (23.5%), compared with TCGA LUAD (11/566, 2.0%) and TCGA HCC (0/372, 0%). Figure 3 summarizes the cross-cohort mutation patterns in HAL versus TCGA LUAD and TCGA HCC.

### Copy-number alterations and tumor mutation burden (TMB)

Copy-number alterations were detected in 4/17 cases (23.5%), comprising *CCND3* amplification (*n* = 2) and single-case events involving *CDKN2A* loss, *MTAP* loss, *SMARCA4* loss, *MYC* amplification, and *TGFBR2* loss. TMB was available for 16/17 cases, with a median of 11.2 mut/Mb (range 0.7–27.7). Using a threshold of ≥ 10 mut/Mb (FDA tissue-agnostic pembrolizumab indication), 9/16 tumors (56.3%) met criteria for TMB-high status [19].

### Metastatic presentation conditional on primary tumor burden

Using the best available T category for each case, the T-stage distribution was T1 in 3/17 (17.6%), T2 in 7/17 (41.2%), T3 in 2/17 (11.8%), and T4 in 5/17 (29.4%) (Table 1). Lymph node metastases were present in 12/17 cases (70.6%), and hematogenous metastases in 9/17 (52.9%). Clinical stage IV tumors typically involved multiple distant metastatic organ-site categories at diagnosis (median 3, range 2–5), most commonly bone, adrenal, liver, and brain.

To contextualize metastatic presentation relative to primary tumor burden, we compared HAL with SEER and TCGA LUAD reference cohorts using T-stratified proportions of metastasis at diagnosis. The primary analytic focus was the combined T1–T2 stratum, in which metastatic presentation was markedly enriched in HAL compared with SEER (HAL 6/10 vs. SEER 486/7015; OR 20.15, 95% CI 5.67–71.65; Fisher *p* = 1.89 × 10⁻⁵) and compared with TCGA (HAL 6/10 vs. TCGA 18/327; OR 25.75, 95% CI 6.67–99.47; Fisher *p* = 1.20 × 10⁻⁵). T1–T4 stratum-specific proportions are summarized descriptively in Table 2.

**Table 2:** Tumor stage of hepatoid adenocarcinomas of lung origin compared with SEER and TCGA of other lung adenocarcinomas cohorts

Cohort	T1 with M1 / total (%)^a e^	T2 with M1 / total (%)	T3 with M1 / total (%)	T4 with M1 / total (%)	T1&T2 with M1 / total (%)
HAL^b^	1/3 (33.3)	5/7 (71.4)	1/2 (50.0)	2/5 (40.0)	6/10 (60.0)
SEER LUAD 2010–2015^c^	172/4217 (4.1)	314/2798 (11.2)	467/1454 (32.1)	616/1027 (60.0)	486/7015 (6.9)
TCGA LUAD^d^	4/110 (3.6)	14/217 (6.5)	1/31 (3.2)	4/18 (22.2)	18/327 (5.5)

a Metastatic at diagnosis was defined as any M1 category within each dataset

b HAL T category reflects best available staging, using pathologic T category for resection specimens and clinical T category for biopsy-only cases, according to AJCC 8th edition criteria

c SEER cases were classified using Derived AJCC 7th edition T and M categories at diagnosis (2010–2015)

d TCGA cases were classified using pathologic PT and PM fields; cases with MX/PMX or uninterpretable T category were excluded

e Percentages are calculated within each T stratum as M1 / (M0 + M1)

Abbreviations: *HAL *hepatoid adenocarcinoma of the lung, *LUAD* lung adenocarcinoma, *SEER* Surveillance, epidemiology, and end results, *TCGA* The cancer genome atlas, *AJCC *American Joint Committee on Cancer, *pT* pathologic primary tumor category, *cT* clinical primary tumor category, *M0/M1* absence/presence of distant metastasis at diagnosis, *MX* metastasis not assessed or unknown

### Outcomes and relationship to stage-conditional metastatic presentation

At last follow-up, 10 patients had died of disease (58.8%), 4 were alive without evidence of disease (23.5%), and 3 were alive with disease (17.6%). By the median follow-up timepoint of 5 months (range, 0.5–103 months), 7 patients were dead of disease (41.2%), 6 were alive with disease (35.3%), and 4 were alive without evidence of disease (23.5%). Median overall survival by Kaplan–Meier analysis was 6 months. Among T1–T2 tumors, 6/10 (60.0%) presented with metastatic disease (M1). Within this T1–T2 subset, early mortality occurred exclusively in patients who were metastatic at diagnosis: 3/6 died within 3 months and 4/6 within 6 months, whereas no deaths occurred within 6 months among non-metastatic T1–T2 cases (0/4).

Within the T1–T2 M1 group, molecular alterations were heterogeneous, including *STK11* in 3/6 (50.0%), *TP53* in 2/6 (33.3%), and *KRAS* in 1/6 (16.7%), along with additional alterations such as *ARID1A/PBRM1*, *SMARCA4*, *BRCA2/NF1*, and *RBM10*. Among T1–T2 M0 cases (*n* = 4), recurrent alterations also involved canonical LUAD pathways, including *STK11* in 3/4 and *KRAS* in 3/4 (with *TP53* in 1/4), together with additional events such as *ATM* and *NF2* alterations and occasional copy-number change (e.g., *TGFBR2* loss).

## Discussion

The term HAL was introduced by Ishikura et al. [2], who recommended two criteria for diagnosis: (i) a conventional acinar or papillary adenocarcinoma pattern, and (ii) a carcinoma component morphologically resembling HCC with associated AFP production. Further investigations have shown that LUADs exhibiting solid-trabecular growth and abundant granular-vacuolated cytoplasm, similar to HCC, commonly carry *STK11* mutations and demonstrate strong HepPar1 immunoreactivity in the absence of other hepatocellular markers [20]. Based on these findings, the authors proposed that isolated HepPar1 expression in LUAD reflects mitochondrial changes driven by *STK11* mutations rather than genuine hepatocellular differentiation, and that such HepPar1–positive solid and granular LUAD represents an undifferentiated variant within this tumor spectrum [20].

The diagnostic evaluation of HAL requires careful integration of morphology, immunophenotype, and anatomic context, as several entities can mimic aspects of its hepatoid phenotype [5–7, 21]. Three differentials are particularly important in routine practice: HCC, combined hepatocellular–cholangiocarcinoma (cHCC-CCA), and conventional non-mucinous LUAD.

###  HAL versus HCC

The HAL–HCC differential remains the most frequently emphasized challenge in the literature [22–24]. Both tumors may show HCC-like cytology with abundant eosinophilic cytoplasm and solid growth. However, HAL often demonstrates features that are not typical of HCC, including complex glandular differentiation and intra- and extracellular mucin. Immunohistochemistry provides decisive support: HAL typically expresses MOC31 and shows negative arginase-1, whereas HCC does not [5]. The presence of mediastinal lymph node metastases also strongly favors a lung primary [25]. This combination of hepatoid morphology with gland formation, mucin, and a lung-type immunophenotype helps anchor the diagnosis of HAL.

###  HAL versus combined hepatocellular–cholangiocarcinoma (cHCC-CCA)

Distinguishing HAL from cHCC-CCA can be particularly difficult in small biopsies, as both may contain hepatocellular-like areas and glandular elements [26–28]. A key discriminator is the immunophenotypic pattern: HAL shows HepPar1, CK7, MOC31, and cytoplasmic TTF-1 expression across both the hepatoid and glandular components, whereas cHCC-CCA typically maintains distinct immunophenotypic identities between its hepatocellular and cholangiocytic elements. Clinical context again assists: a solitary lung mass with mediastinal nodal involvement is far more consistent with HAL than with a metastatic cHCC-CCA.

### HAL versus conventional non-mucinous LUAD

The differential also includes conventional non-mucinous LUAD, especially when hepatoid features are focal. While non-mucinous LUAD can show solid growth or eosinophilic cytoplasm, the triad of HCC-like cytology, complex glandular architecture, and mucinous differentiation is unusual for this group. Immunophenotype again provides clarity: non-mucinous LUAD typically shows nuclear TTF-1 positivity and is HepPar1 negative, whereas HAL demonstrates cytoplasmic TTF-1 and HepPar1 positivity, reflecting its hepatoid differentiation.

Taken together, these distinctions highlight that HAL occupies a unique diagnostic space characterized by hepatocellular-like morphology blended with glandular and mucinous features, supported by a hybrid immunophenotype that differs from both hepatic tumors and conventional LUAD. In routine practice, clinicoradiologic correlation combined with a focused immunohistochemical panel—typically TTF-1, HepPar1, CK7, and MOC31—provides a reliable and pragmatic approach to resolving this differential, even in limited biopsy material.

At the molecular level, our cohort aligned more closely with established LUAD biology than with a distinct hepatoid-specific genotype. Recurrent alterations involved canonical LUAD pathways, most prominently *STK11* and *KRAS*, with additional events affecting oxidative-stress response (*KEAP1*) and chromatin-remodeling programs (*SMARCA4*). These alterations collectively situate HAL within recognized high-risk LUAD molecular landscapes, consistent with its aggressive clinical behavior [14, 15]. Importantly, low-burden metastatic cases did not converge on a single defining genotype. Within the T1–T2 M1 subset, *KRAS/STK11/TP53* combinations were not universal; instead, alterations spanned diverse biological programs, including chromatin remodeling and DNA-damage response (*ARID1A/PBRM1*, *SMARCA4*, *BRCA2/NF1*, *RBM10*). Conversely, T1–T2 M0 cases also frequently harbored canonical LUAD drivers—most notably *STK11* and *KRAS*—highlighting that these alterations alone are insufficient to account for early dissemination. Taken together, these observations argue against a single “signature genotype” as a prerequisite for early metastasis. Rather, they support a convergent model in which hepatoid differentiation can arise across multiple LUAD genomic backgrounds, potentially reflecting a progression-associated program accessible through different high-risk molecular routes [29–32].

*STK11* mutations were the most frequent genetic alterations identified in HAL. Most *STK11*-mutated HALs displayed a predominantly solid growth pattern composed of tumor cells with abundant granular or vacuolated/clear cytoplasm, accompanied by HepPar1 positivity and cytoplasmic TTF-1 expression. Rare *STK11*-mutated HALs showed mixed patterns typical of lung adenocarcinoma, such as acinar or papillary architecture, without a solid component (Table S4).

A recent comprehensive study reported similar findings, demonstrating that *STK11* mutations or loss, together with HepPar1 expression, correlate with marked mitochondrial accumulation, which underlies the characteristic granular eosinophilic cytoplasm [20]. The authors proposed that HepPar1–expressing LUADs represent a spectrum of *STK11*-mutated tumors, ranging from better-differentiated forms (predominantly acinar, variable nuclear TTF-1 positivity) to poorly differentiated forms (solid, lacking nuclear TTF-1 expression), with the latter showing the most pronounced granular features [20].

None of the HALs with *SMARCA4* mutations in the present study exhibited undifferentiated or rhabdoid morphology, features typically associated with thoracic *SMARCA4*-deficient undifferentiated tumors.

HAL appears molecularly distinct from both HCC and cHCC-CCA. Large-scale genomic studies of HCC consistently identify *TP53*, *TERT* promoter, and *CTNNB1* as the most frequently mutated genes, reflecting canonical hepatocarcinogenesis pathways [28, 33]. In contrast, cHCC-CCA demonstrates a hybrid molecular profile, with alterations characteristic of either intrahepatic cholangiocarcinoma or HCC—including *TERT*, *TP53*, *KRAS*, and *ARID1A*—depending on the dominant lineage represented [26]. Recent work has further refined the molecular taxonomy of cHCC-CCA by identifying distinct *NOTCH*-signaling subgroups with prognostic significance [27]. These *NOTCH*-defined classes underscore the developmental and lineage-plasticity programs active in cHCC-CCA. Notably, such *NOTCH*-driven subgroups have not been observed in HAL, reinforcing that HAL does not share the molecular architecture of mixed-lineage hepatic tumors. Taken together, these comparisons highlight that HAL occupies a LUAD-aligned molecular space, rather than representing a variant of hepatocellular or mixed hepatocellular–cholangiocytic neoplasia. This distinction is diagnostically meaningful, particularly when hepatoid morphology raises concern for a primary hepatic tumor.

Our findings support a reinterpretation of HAL’s reputed “aggressiveness,” showing that the defining feature is a heightened likelihood of metastatic presentation at comparable primary tumor burden, not an intrinsic aggressiveness inferred from stage distributions alone. Although prior reports consistently characterize HAL as aggressive, these impressions arise largely from small, referral-enriched cohorts in which stage mix is difficult to interpret [6, 7, 10, 14, 15, 34–38]. By examining metastatic-at-diagnosis rates within T-category strata and benchmarking against SEER and TCGA LUAD references, our data show that HAL remains enriched for metastatic presentation even when restricted to comparable T1–T2 burden. This burden-conditioned approach provides a more stable interpretive denominator and may help harmonize findings across institutions, particularly for rare variants where absolute stage distributions and ascertainment patterns vary substantially.

Survival observations in rare tumors are inherently constrained by small numbers, stage imbalance, and heterogeneous therapies, limiting the interpretability of cross-cohort comparisons [6, 7]. Within this context, our outcome data are best viewed as supportive of the same burden-conditioned metastatic signal rather than as an independent survival analysis. When aligned to primary tumor burden, early deaths clustered among low-burden cases that were metastatic at presentation, whereas low-burden non-metastatic cases did not exhibit early mortality. This pattern parallels the clinical gestalt described in pooled reviews and reinforces the notion that metastatic presentation at low burden, rather than stage mix alone, captures the biologic signature that has historically been labeled as “aggressiveness” in HAL.

Limitations of this study largely reflect cohort size and real-world heterogeneity. Small numbers preclude definitive stage-adjusted survival analyses or genotype–outcome modeling, and reliance on best-available T staging introduces the degree of variability typical of routine practice [6, 7]. SEER and TCGA differ in staging ascertainment and case mix, so our emphasis is on directionally consistent, burden-conditioned comparisons rather than incidence estimation.

These constraints point directly toward practical next steps. Multi-institution cohorts applying a shared burden-conditioned framework are needed to determine whether the disproportionate metastatic-at-diagnosis signal persists across practice settings. On the biologic side, assembling larger cohorts with harmonized genomic data and integrated transcriptomic/epigenetic profiling will allow testing whether T1–T2 M1 cases share reproducible pathway-level programs—such as immune-evasion, oxidative-stress response, or chromatin-remodeling signatures—distinct from T1–T2 M0 cases despite similar driver alterations. Together, these directions pair a realistic diagnostic approach with a burden-conditioned clinical framework that can standardize future studies and refine counseling for patients with HAL.

HAL must be recognized because it occupies a diagnostically consequential intersection of morphology, immunophenotype, anatomic context, and molecular identity. Its hepatocellular-like features create immediate risk for misclassification as metastatic HCC or cHCC-CCA, diagnoses that carry fundamentally different staging, treatment pathways, and prognostic expectations. Accurate identification therefore prevents inappropriate attribution of a lung primary to occult liver disease, avoids delays in lung-directed therapy, and anchors clinical decision-making to the correct organ of origin.

From a pathology standpoint, HAL also demands recognition because it expands the differential for hepatoid morphology beyond hepatic primaries and underscores the need for integrated interpretation of morphology, hepatic and pulmonary markers, and anatomic distribution. Its consistent expression of adenocarcinoma markers, absence of hepatocyte-restricted markers such as arginase-1, and LUAD-aligned genomic architecture collectively reinforce that HAL is a LUAD with hepatoid differentiation—not a hepatic tumor masquerading in the thorax.

Finally, recognizing HAL has emerging clinical relevance [39–41]. Its burden-conditioned metastatic propensity, distinct from raw stage mix, suggests a biologic behavior that warrants attention in counseling and follow-up. As larger cohorts refine its molecular and pathway-level signatures, accurate classification will be essential for interpreting outcomes, harmonizing research across institutions, and identifying potential therapeutic vulnerabilities.

## Supplementary Information

Below is the link to the electronic supplementary material.

Supplementary Material 1 (DOCX 4.90 MB)

## Data Availability

The data generated in this study are available from the corresponding author upon reasonable request.
