# Supplementary material for: Hepatoid adenocarcinoma of the lung: clinicopathologic and molecular analysis of 17 cases
Source: Virchows Arch. 2026 May 7;489(1):91–101. doi: 10.1007/s00428-026-04558-3 (PMC13368951; doi:10.1007/s00428-026-04558-3)
Supplement: Supplementary file 1 — Supplementary Material 1 (DOCX 4.90 MB) [file 428_2026_4558_MOESM1_ESM.docx]

**Supplemental Material**

**Immunohistochemistry**
Immunohistochemistry (IHC) was performed primarily as part of routine diagnostic workup at diagnosis, with marker selection varying by tissue availability and the contemporaneous differential diagnosis. Additional stains were performed retrospectively on available material in a subset of cases to improve comparability and better align with commonly used panels in the HAL–HCC differential. Results were abstracted from pathology reports and/or slide review. Positivity rates are reported as n positive/n tested, with unperformed stains recorded as not available. IHC was performed on 3-µm paraffin sections using a fully automated staining platform (Benchmark XT System, Roche Tissue Diagnostics, Tucson, AZ). Antibodies included AE1/AE3 (1:200, Leica), MOC31, PD-L1 (SP263), p40 (BC28), TTF-1 (SP141), Napsin A (MRQ-60), CK7 (SP52), HepPar1 (OCH1E5), AFP (43-14A), and Arginase-1 (SP156); non-Leica antibodies were prediluted Roche reagents. Programmed death-ligand 1 (PD-L1) tumor proportion score (TPS; percent of viable tumor cells with membranous staining) was abstracted from pathology reports.

**Surveillance, Epidemiology, and End Results (SEER) LUAD comparator cohort**
A population-based LUAD comparator cohort (2010–2015) was constructed in SEERStat (Frequency Session), restricted to lung and bronchus primaries with malignant behavior, microscopic confirmation, and adenocarcinoma histology (ICD-O-3 8140, 8250–8255). Derived AJCC 7th edition T and M variables at diagnosis were used. Metastatic status was defined as any M1 (M1a/M1b/M1 NOS). Denominators were defined as M0 + any M1 within each T stratum, excluding cases with uninterpretable or missing metastatic category; stratum counts were obtained from the exported SEERStat T×M frequency table.

**The Cancer Genome Atlas (TCGA) LUAD comparator cohort**
TCGA LUAD clinical staging data were obtained from cBioPortal (TCGA Firehose Legacy LUAD cohort) using clinical attributes AJCC_TUMOR_PATHOLOGIC_PT and AJCC_METASTASIS_PATHOLOGIC_PM. T categories were collapsed to T1–T4 and metastatic status defined as any M1. Analyses included cases with interpretable T and known M category; cases annotated as MX were excluded and “percent eligible” reflects this restriction.

**Statistical analysis**
Descriptive statistics were used to summarize clinicopathologic features, immunophenotypes, and molecular alterations. For the metastatic-at-diagnosis comparison, between-cohort hypothesis testing was performed using Fisher’s exact test within the prespecified T1–T2 combined stratum. Effect size was summarized as an odds ratio (OR) with 95% confidence interval. Overall survival (OS) was defined as time from diagnosis to death from any cause or last follow-up and summarized using the Kaplan–Meier method. Median survival was summarized for the overall cohort and for stage IV cases. Early mortality at 3 and 6 months was reported to provide a robust near-term outcome metric given variable follow-up and treatment heterogeneity. Given limited sample size, treatment heterogeneity, and non-uniform staging ascertainment across specimen types, molecular–outcome associations were treated as descriptive rather than inferential. Analyses were performed in Python using lifelines, scipy/statsmodels, and matplotlib. A two-sided p value <0.05 was considered statistically significant for the prespecified comparison.

## **DNA extraction**

Genomic DNA was extracted from FFPE tissue sections using the Bionano Ionic FFPE to DNA isolation kit (Bionano Genomics, San Diego, CA).

## **Next-generation sequencing (NGS) and copy number analysis**

Targeted NGS was performed for 17 cases using the Personal Genome Diagnostics (PGDx™) elio™ tissue complete assay, an FDA-approved comprehensive genomic profiling platform (PGDx, Baltimore, MD, USA). This panel interrogates 505 genes for single-nucleotide variants (SNVs), insertions and deletions (indels), gene rearrangements, and amplifications, as well as complex genomic signatures including tumor mutational burden (TMB, muts/Mb, exome equivalent) and microsatellite instability (MSI) status. Methods followed previously described protocols. In brief, DNA extractions were performed ensuring ≥20% viable tumor nuclei, with input DNA of 100 ng recommended (validated down to 50 ng). Library preparation involved mechanical shearing to ~200 bp fragment size, end repair, adapter ligation, PCR amplification, bead-based cleanups, and QC via fragment analyzer. Hybrid capture was performed targeting full exons and selected introns of the 505 genes. Sequencing was conducted on the Illumina NextSeq platform (NextSeq-550Dx), and data was processed through the standard PGDx bioinformatics pipeline.

For one case (Case #2), the Oncomine Precision Assay (Thermo Scientific, Waltham, MA) was performed using Ion Torrent technology. This kit interrogates mutations, copy numbers, and fusions across 50 cancer-relevant genes. Sequencing data were aligned and variants called using on-instrument Genexus software, with annotation performed in Pathology Workbench (GenomOncology, Cleveland, OH).

Chromosomal microarray analysis (CMA) was performed for all study cases as part of the clinical diagnostic workup using the Affymetrix OncoScan CNV array platform (Affymetrix, ThermoFisher Scientific), following the manufacturer’s protocol. Data were analyzed with the Affymetrix Chromosome Analysis Suite software and reviewed by two cytogeneticists (M.S. and X.L.), with manual review of all identified copy number alterations and stretches of copy-neutral loss of heterozygosity (CNLOH).


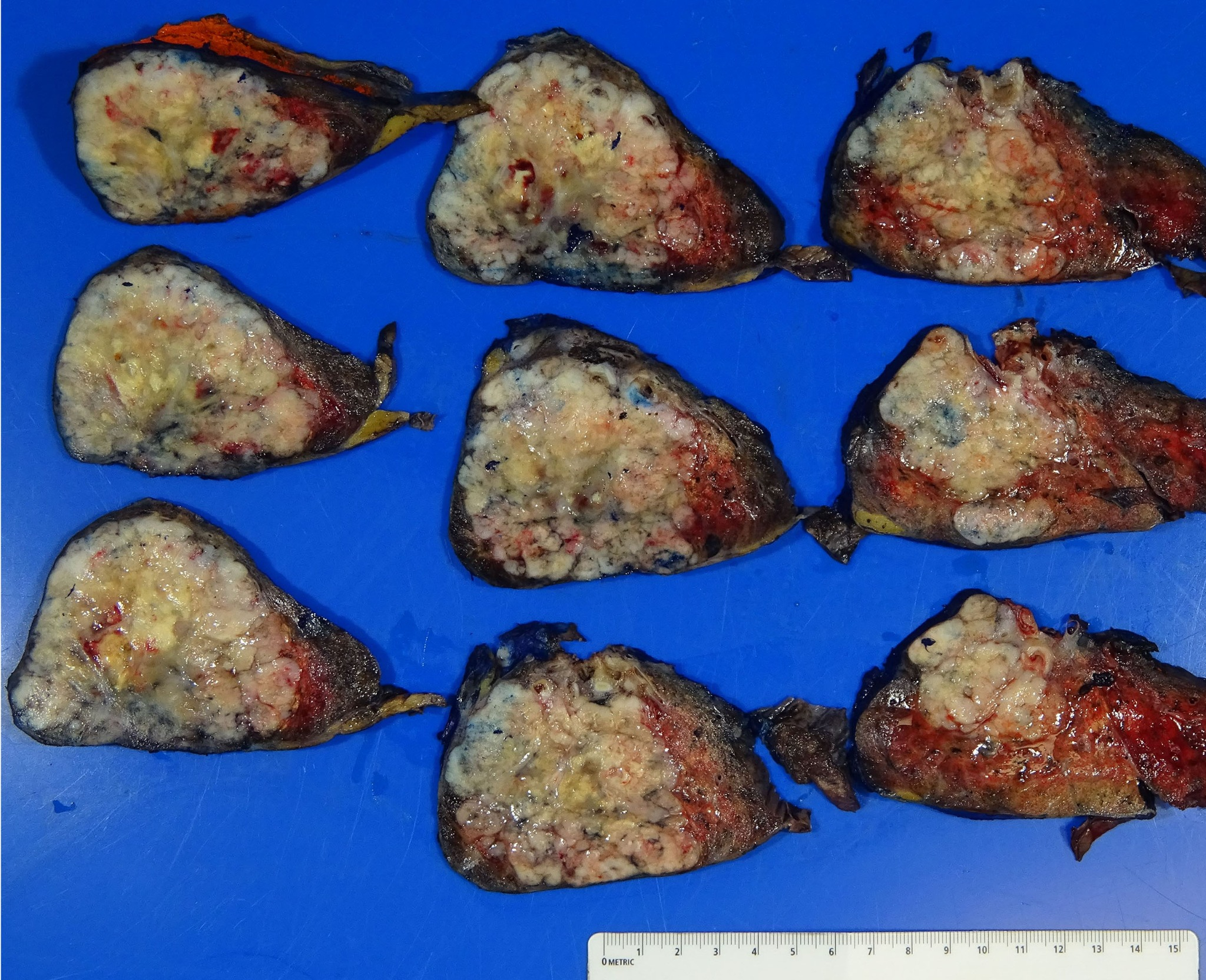


**Figure S1 Gross appearance of hepatoid adenocarcinoma of the lung**
Serial sections of the resected lung show a large, poorly circumscribed tan–yellow mass with a variegated cut surface composed of soft, friable tissue, extensive coagulative and geographic necrosis, focal hemorrhage, and areas of waxy–greasy consistency typical of hepatoid differentiation. The tumor effaces the underlying lung parenchyma and occupies the majority of the sectioned lobe.

**Table S1** Individual demographic characteristics of patients with hepatoid adenocarcinoma of the lung (HAL)

| **Patient** | **Age at diagnosis** | **Gender** | **Smoking history** | **Pack year** | **Pulmonary location** | **Tumor size (cm)** |
| --- | --- | --- | --- | --- | --- | --- |
| **1** | 64 y, 7 m | F | Former | 36 | LUL (Left lingula) | 1.2 |
| **2** | 62 y, 4 m | F | Former | 30 | RUL | 2.1 |
| **3** | 72 y, 0 m | M | Former | 8 | LUL | 1.7 |
| **4** | 66 y, 7 m | F | Former | 100 | RUL | 7.8* |
| **5** | 67 y, 0 m | F | Former | 25 | RLL, RML | 6.2 |
| **6** | 71 y, 1 m | M | Former | 38 | RML | 1.9 |
| **7** | 81 y, 8 m | F | Former | 60 | LUL | 7.2* |
| **8** | 79 y, 11 m | F | Former | 25 | RUL, RLL | 7.7 |
| **9** | 61 y, 6 m | M | Never | NA | RML | 6.3* |
| **10** | 68 y, 11 m | M | Former | 47 | RUL | 7.7 |
| **11** | 79 y, 6 m | M | Never | NA | RML | 1.8* |
| **12** | 67 y, 1 m | M | Former | 41 | RUL | 4.8* |
| **13** | 50 y, 9 m | F | Never | NA | LLL | 2.1 |
| **14** | 68 y, 7 m | M | Former | 78.6 | RLL | 4.0* |
| **15** | 73 y, 10 m | F | Never | NA | RUL | 4.3* |
| **16** | 84 y, 11 m | F | Former | 50 | LUL | 4.5* |
| **17** | 87 y, 4 m | F | Former | 63 | RLL | 4.5* |

*These tumor size values indicate the largest dimension estimated from imaging rather than gross pathologic measurement.
Abbreviations: LUL, left upper lobe; LLL, left lower lobe; RUL, right upper lobe; RML, right middle lobe; RLL, right lower lobe.

**Table S2** Clinical follow-up of HAL patients

| **Patient** | **Specimen type** | **Stage^a^** | **Vital status^b^** | **Follow-up^c^ (months)** | **Sites of disease^d^** | **Treatment summary^e^** |
| --- | --- | --- | --- | --- | --- | --- |
| **1** | Resection | pT1bN2Mx, cIII | DOD | 12 | Lymph nodes | 1. Chemo |
| **2** | Resection | pT1cN0Mx, cI | NED | 18 | None | None |
| **3** | Resection | pT2aN1M1c, cIV | DOD | 6 | Lymph node, adrenal, and spine | 1. Chemo 2. Palliative RT |
| **4** | Biopsy | cT4N1M1c, cIV | DOD | 1 | Lymph node, adrenal, skull | 1. Chemo + RT |
| **5** | Resection | pT4N1Mx, cIII | NED | 103 | Lymph node | 1. Chemo  2. Chemo |
| **6** | Resection | pT2aN0Mx, cI | NED | 11 | None | None |
| **7** | Biopsy | cT3NxMx, cIII | DOD | 1 | None | None |
| **8** | Biopsy | cT4N3Mx, cIII | DOD | 5 | Lymph node | 1. Chemo + RT 2. IO |
| **9** | Biopsy | cT4N1M1b, cIV | AWD | 7 | Lymph node, liver | 1. Chemo + IO |
| **10** | Resection | pT4N0Mx, cIIIB | DOD | 32 | None | 1. Chemo + RT 2. Chemo 3. RT 4. Chemo + IO 5. Chemo |
| **11** | Biopsy | cT1bN2M1c, cIV | AWD | 1 | Lymph nodes, pelvis, femur, spine | 1. Chemo + RT |
| **12** | Biopsy | cT2bN2M1b, cIV | AWD | 2 | Lymph nodes, spine | None |
| **13** | Resection | pT2N0M0, cI | NED | 7 | None | None |
| **14** | Biopsy | cT2aN2M1c, cIV | DOD | <1 | Lymph nodes, adrenal, spine, liver | Hospice/Palliative |
| **15** | Biopsy | cT2bN2M1b, cIV | DOD | <1 | Lymph nodes, brain | Hospice/Palliative |
| **16** | Biopsy | cT2bN2M1c, cIV | DOD | 3 | Lymph nodes, adrenal, rib | 1. Chemo |
| **17** | Biopsy | cT3N2M1c, cIV | DOD | <1 | Lymph nodes, spine | Hospice/Palliative |

ᵃ Stage at presentation is reported using pathologic TNM/category for resection specimens and clinical TNM/category for biopsy-only specimens.
ᵇ Vital status at last follow-up is reported as no evidence of disease, alive with disease, or died of disease.
ᶜ Follow-up time is measured from diagnosis to death or last documented follow-up.
ᵈ Sites of disease represent clinically and/or pathologically documented sites of disease spread.
ᵉ Treatment summary includes systemic and local therapies administered during the disease course; sequential numbering indicates changes in treatment regimen over time rather than repeated cycles of the same regimen.

**Abbreviations**

**HAL**, hepatoid adenocarcinoma of the lung; **NED**, no evidence of disease; **AWD**, alive with disease; **DOD**, died of disease; **TNM**, tumor-node-metastasis; **p**, pathologic; **c**, clinical; **Chemo**, chemotherapy; **IO**, immune checkpoint inhibitor therapy; **RT**, radiotherapy.

**Table S3 Immunohistochemical staining results in HAL cases**

| **Case** | **MOC31** | **CK AE1/AE3** | **CK7^a^** | **TTF-1^b^** | **HepPar1** | **p40** | **Napsin A** | **AFP^c^** | **Arginase-1** |
| --- | --- | --- | --- | --- | --- | --- | --- | --- | --- |
| **1** | **+** | NA | + (LN) | + | + | NA | + (LN) | NA | NA |
| **2** | **+** | NA | + | + | + | - | NA | NA | NA |
| **3** | **+** | + (LN) | NA | + (subset) | + | + (subset) | NA | NA | NA |
| **4** | **+** | + | NA | + | + | - | NA | NA | + |
| **5** | **+** | NA | NA | Weak | + | NA | NA | NA | NA |
| **6** | **+** | NA | + | + | + | NA | NA | NA | NA |
| **7** | **+** | NA | NA | + | + | NA | NA | NA | NA |
| **8** | **+** | + | + | + | + | - | - | - | NA |
| **9** | **+** | NA | NA | + | + | NA | - | - (serum) | - |
| **10** | **+** | NA | + | + | + | - | - | NA | NA |
| **11** | **+** | + | + | + | + | - | - | NA | - |
| **12** | NA | + | + | + | + | - | - | NA | NA |
| **13** | **+** | NA | + | + | + | NA | + | NA | NA |
| **14** | **+** | + | + | + | + | NA | - | NA | NA |
| **15** | **+** | + | + | + | + | - | - | - | - |
| **16** | **+** | NA | + | + | + | - | - | - | - |
| **17** | **+** | NA | + | + | + | NA | + | + | - |
| NA= Not available, + Positive, - Negative. | | | | | | | | | |

ᵃ Parenthetical site designations indicate staining performed on a metastatic site rather than the primary tumor (eg, lymph node).
ᵇ TTF-1 results refer to both **cytoplasmic and nuclear** staining, with cytoplasmic being the predominant pattern.
ᶜ For AFP, “− (serum)” indicates negative serum AFP rather than negative tumor immunohistochemical staining.

**Abbreviations**
**HAL**, hepatoid adenocarcinoma of the lung; **NA**, not available; **+**, positive; **−**, negative; **LN**, lymph node; **TTF-1**, thyroid transcription factor 1; **AFP**, alpha-fetoprotein.

**Table S4** **Variants of known and uncertain significance, tumor mutational burden, and immunotherapy-related biomarkers in HAL**

| **Case** | **VKS^a^** | **VUS^b^** | **Histologic Patterns** | **CNV^c^** | **TMB^d^** | **PD-L1^e^** |
| --- | --- | --- | --- | --- | --- | --- |
| 1 | KRAS p.G12C (7%) STK11 p.P281fs (12%) TP53 p.G154S (8%) LRP1B p.E4499* (7%) | ABL2 p.S127T (8%) ATRX p.T836N (6%) BCL2L2 p.W144C (11%) CD276 p.G130S (51%) EP300 p.Y1089F (12%) ERCC3 p.R198L (8%) FLT4 p.C93* (8%) GPR124 p.G1062S (48%) GPR124 p.G1246V (44%) KEAP1 p.G509V (11%) MST1R p.G1303R (48%) PIK3CG p.T330K (9%) PRKDC p.D3180Y (8%) RBM10 p.R817L (7%) SLIT2 p.I450T (8%) WHSC1 p.Q63L (8%) WT1 p.R19L (12%) | acinar (50%) papillary (40%) micropapillary (10%) | None | 20.8 | <1% |
| 2 | KRAS p.G12V (25%) |  | solid (80%) acinar (10%)  complex glands (10%) | None | NA | <1% |
| 3 | CDKN2A p.D108Y (24%) TP53 p.R175H (35%) DNMT3A svLOF (7%) KMT2D p.E4650* (17%) | ABL2 p.R198* (8%) BCOR p.D1389N (30%) ERCC3 p.P110S (5%) KEAP1 p.E488K (20%) KMT2C p.E4008D (18%) RBM10 p.E215Q (35%) TRAF7 p.S108L (18%) | solid (65%)  complex glands (15%) lepidic (10%)  micropapillary (5%) squamous cell carcinoma (5%) | None | 9.2 | <1% |
| 4 | HRAS p.E49K (39%) KEAP1 p.R470C (36%) STK11 p.D194N (39%) TP53 p.P75fs (69%) | ALK p.K507N (14%) BCORL1 p.E1255K (38%) BRCA2 p.V1306A (9%) CUL3 p.E386K (14%) H3F3C p.L70M (62%) H3F3C p.H39D (12%) HIST1H1C p.A151D (38%) KMT2C p.D2324E (17%) KMT2C p.D1222N (36%) KRAS p.K104M (30%) LRP1B p.G1908V (14%) NCOR1 p.E632del (23%) SMARCA4 p.S699F (37%) STK11 p.E291Q (37%) STK11 p.E351Q (34%) STK11 p.E357Q (34%) TAF1 p.E1513Q (35%) WHSC1L1 p.E592K (15%) | solid (100%) | None | 22.3 | 50% |
| 5 | KRAS p.G12D (19%) SMARCA4 p.R1005* (16%) STK11 p.D53fs (22%) | ALK p.S289Y (13%) AMER1 p.N541K (12%) GRM3 p.N348K (11%) GRM3 p.T410S (12%) KMT2C p.N4848S (52%) KMT2D p.G1010W (23%) MTAP p.R235Q (47%) NUP93 p.M814T (47%) PIK3C2B p.R531V (11%) SDHA p.R5W (48%) SNCAIP p.Q532K (11%) SPTA1 p.D1378Y (15%) | solid (30%) complex glands (30%) acinar (20%) mucinous (10%) papillary (5%) lepidic (5%) | None | 14.6 | 1% |
| 6 | KRAS p.G12D (16%) NF2 p.L49fs (11%) STK11 p.V66fs (14%) | CYLD p.G589W (10%) DDR1 p.R276Q (5%) FANCM p.E1610* (7%) FLT1 p.V112E (7%) IRS2 p.R554C (6%) NOTCH3 p.D561N (42%) PDGFRB p.Y686C (45%) POT1 p.S193F (10%) | complex glands (45%) solid (30%) acinar (15%) micropapillary (5%) lepidic (5%) | None | 7.7 | 30% |
| 7 | ARID2 p.E108* (20%) PIK3R3 p.Q284* (19%) TP53 p.K321fs (31%) | AKT1 p.T211I (21%) AR p.D297N (18%) ARAF p.R479H (17%) BRCA1 p.Q1281H (30%) HDAC6 p.Q690H (18%) KDM5C p.L1560F (22%) NOTCH3 p.D941V (56%) PIK3CG p.V188M (52%) | solid (60%) complex glands (20%) acinar (20%) | None | 8.5 | 1% |
| 8 | KRAS p.G12C (29%) TP53 p.R273C (41%) TERT svLOF (29%) | ALK p.P294T (25%) AMER1 p.E905D (16%) ARID1B p.G466C (39%) ATRX p.G1290V (14%) FANCA p.R609I (32%) GRIN2A p.H702D (23%) KEAP1 p.N222I (50%) LRP1B p.C1531S (30%) NTRK3 p.T777R (14%) PAX5 p.S339T (29%) PAX5 p.Q57H (16%) PDGFRA p.E495* (29%) PRKN p.N313I (40%) PTPRD p.G251E (30%) TLR4 p.S71T (29%) | solid (100%) | None | 11.5 | >50% |
| 9 | ERCC2 p.F568fs (55%) | FANCB p.A149T (100%) FLT4 p.L295R (17%) SPEN p.A3262_P3263del (39%) TET2 p.A1512E (21%) | solid (50%) acinar (40%) lepidic (10%) | None | 6.9 | 1% |
| 10 | ATR p.R796* (45%) CDKN2A p.D108G (66%) KEAP1 p.R204fs (60%) SMARCA4 p.S1382* (65%) TP53 p.C242F (68%) LRP1B p.L4186fs (52%) | ABL2 p.G883A (57%) CSF1 p.E322V (64%) CTLA4 p.V170L (39%) CYLD p.L297V (12%) EPHB4 p.E697D (7%) ESR1 p.R158P (56%) GLI1 p.P175Q (12%) JAK1 p.C752R (64%) KEL p.R47P (53%) LZTR1 p.L555V (83%) MAP3K1 p.M641V (15%) MITF p.T186R (61%) NOTCH1 svLOF (63%) NOTCH3 p.K1940fs (23%) NUTM1 p.L200I (45%) PDPK1 p.G403D (16%) PTCH1 p.R1113C (20%) RANBP2 p.G2517V (66%) SMAD3 p.Q242L (51%) TYRO3 p.L821fs (15%) | solid (70%) acinar (20%) mucinous (10%) | None | 27.7 | <1% |
| 11 | BRCA2 p.S1982fs (59%) NF1 c.4577+1_4577+5del (10%) NF1 p.G1998* (9%) | FAT1 p.E4440G (11%) KMT2A p.R130P (13%) LATS1 p.P265S (12%) | solid (100%) | None | 5.4 | <1% |
| 12 | AKT1 p.E17K (3%) BRAF p.G469V (5%) SMARCA4 c.356-1G>T (5%) | ABRAXAS1 p.S310N (47%) ARAF p.K339Q (9%) ARID1A p.D1116E (51%) ARID2 p.S659P (50%) CREBBP p.P880S (52%) GATA1 p.P294L (8%) MAP2K2 p.A176T (49%) | solid (100%) | None | 10.8 | 5% |
| 13 | ATM p.T1284fs (54.9%) STK11 p.L228fs (12.5%) |  | acinar (60%) papillary (30%) mucinous (10%) | None | 0.7 | 5% |
| 14 | APC p.E1451* (28%) ARID1A p.D734fs (30%) PBRM1 p.Q431fs (31%) STK11 p.G56fs (28%) LZTR1 p.E451* (32%) | ASXL1 p.S903L (48%) DDR1 p.R276Q (32%) EPHA3 p.V506L (34%) FANCL p.V208A (48%) FLT1 p.R281L (28%) KEAP1 p.W252C (33%) PDGFRA p.K627N (21%) PIK3C2B p.A234S (51%) RANBP2 p.I154V (53%) SMAD2 p.F206L (30%) VHL p.L116R (35%) | acinar (30%) complex glands (20%) papillary (20%) solid (20%) mucinous (10%) | TGFBR2 loss (0) | 16.2 | <1% |
| 15 | KRAS p.G12C (34%) STK11 p.Q152* (38%) DNMT3A p.Y660C (6%) | ARID1B p.D1415G (51%) HGF p.I691L (29%) RICTOR p.Q506E (21%) | solid (100%) | CDKN2A loss (0) MTAP loss (0) MYC amp (22) SMARCA4 loss (0) | 8.5 | 5% |
| 16 | RBM10 p.I303fs (32%) STK11 p.R40fs (39%) TP53 p.T155I (48%) TP53 p.V157L (49%) | CARD11 p.R423Q (24%) EPHA7 p.G687W (26%) ERBB2 p.H809R (51%) ERCC6 p.G444A (21%) FGF14 p.P203T (20%) GATA1 p.T130K (18%) IGF2R p.Q2055H (30%) IRS2 p.G1226W (32%) KAT6A p.V1486F (23%) KEAP1 p.Y572C (28%) PDGFRA p.M734I (17%) PIK3C2B p.G143V (27%) PIK3CG p.A797V (46%) POLE p.N595I (26%) RET p.R297H (50%) SPTA1 p.F1123L (18%) TLR9 p.R337C (8%) TSHR p.P168L (24%) NTRK3 c.465-1_465delGGinsTT (22%) | solid (100%) | CCND3 amp (6) | 23.1 | 5% |
| 17 | BRAF p.G469V (22%) KEAP1 p.Y345* (30%) MYC p.P260A (14%) RBM10 p.W658* (23%) STK11 p.Q137* (31%) | HSD3B1 p.Y270H (14%) JAK1 p.L540V (55%) MST1R p.W837C (22%) PIK3R3 p.S23L (24%) RICTOR p.R1110H (50%) TSC2 p.E1344K (49%) | solid (60%) complex glands (40%) | None | 12.3 | <1% |

ᵃ VKS denotes variants of known significance, including pathogenic and likely pathogenic alterations. Variant allele frequencies are shown in parentheses.
ᵇ VUS denotes variants of uncertain significance. Variant allele frequencies are shown in parentheses.
ᶜ CNV denotes copy-number variation; values in parentheses indicate copy number. “None” indicates that no copy-number alteration was reported.
ᵈ TMB is reported as mutations per megabase.
ᵉ PD-L1 is reported as tumor proportion score (TPS).

**Abbreviations**
**HAL**, hepatoid adenocarcinoma of the lung; **VKS**, variants of known significance; **VUS**, variants of uncertain significance; **CNV**, copy-number variation; **TMB**, tumor mutational burden; **PD-L1**, programmed death-ligand 1; **TPS**, tumor proportion score; **svLOF, alteration annotated as predicted loss of function**.
